# Past, Present, and Future of Hyperthermic Intraperitoneal Chemotherapy (HIPEC) in Ovarian Cancer

**DOI:** 10.7759/cureus.15563

**Published:** 2021-06-10

**Authors:** Mona Mishra, Nilanchali Singh, Prafull Ghatage

**Affiliations:** 1 Obstetrics and Gynecology, Maulana Azad Medical College, New Delhi, IND; 2 Obstetrics and Gynecology, All India Institute of Medical Sciences, New Delhi, IND; 3 Gynecology and Oncology, Tom Baker Cancer Center, Calgary, CAN

**Keywords:** hipec, hyperthermic intraperitoneal chemotherapy, ovarian cancer, clinical trials, technique

## Abstract

Hyperthermic intraperitoneal chemotherapy (HIPEC), along with optimal cytoreductive surgery, has been debated to be a viable option for the treatment of advanced epithelial ovarian cancer with peritoneal carcinomatosis. HIPEC is associated with a direct and improved penetration of chemotherapy drugs into the affected tissue and is associated with fewer systemic side effects. There is no standard protocol for the use of HIPEC in advanced ovarian cancer. Hence, there is controversy over the timing, dose, duration, and efficacy of HIPEC. In this review, the history, technique, current evidence, recommendations, and future directions of HIPEC are discussed.

## Introduction and background

**Figure 1:**
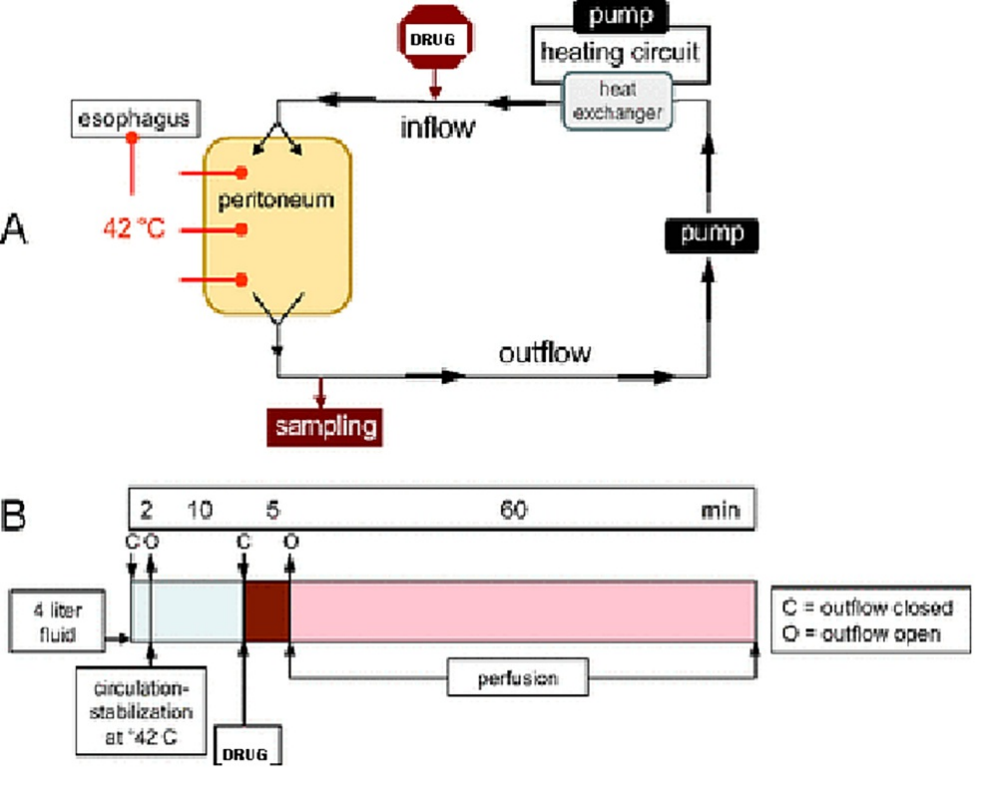
Diagrammatic representation of HIPEC HIPEC: hyperthermic intraperitoneal chemotherapy

Ovarian malignancy is the most lethal of all gynecological cancers. The standard of care for ovarian cancer is surgery in early stages and platinum-based chemotherapy followed by interval debulking surgery in advanced cases. With this norm followed, the five-year survival is less than 30% and recurrence rates are high. Ovarian cancer is known for local spread to the peritoneum, hence, local therapy seems promising. Intraperitoneal (IP) chemotherapy was developed with this idea, and a systemic review found its results promising, with increased survival time and reduced risk of mortality by 12% with each cycle of intraperitoneal therapy [1]. However, the therapy could not gain popularity due to the higher incidence of adverse events. Lately, increased temperature of the chemotherapy agent used intra-peritoneally is being evaluated for its efficacy in ovarian malignancy. Hyperthermic intraperitoneal chemotherapy, popularly called hyperthermic intraperitoneal chemotherapy (HIPEC), is a technique for delivering a chemotherapeutic agent, in which a heated solution of chemotherapy agent is perfused throughout the peritoneal space (Figure 1). It has been used for the treatment of advanced peritoneal malignancies, including gastrointestinal (colorectal and appendiceal) and advanced ovarian cancers. The aim is to target residual disease after cytoreductive surgery (CRS) by directly acting on the cancer cells present on the peritoneal surface. The blood peritoneal barrier limits systemic absorption of the chemotherapy agent, hence reducing its side effects and toxicity. HIPEC has a controversial role in the management of ovarian cancers. This review deals with the historical aspects, current role, and future perspective of HIPEC.

## Review

Rationale of hyperthermia

Hyperthermia has a multi-fold effect on cancer cells. Firstly, heat encompasses a direct cytotoxic effect on cancer cells by the increased production of lysosomes. Secondly, heat has a synergistic effect with certain anti-mitotic agents (cisplatin, paclitaxel, oxaliplatin, and mitomycin) and potentiates their action [2]. Thirdly, heat improves the penetration of chemotherapy, leading to the increased sensitivity of tumor cells to chemotherapy and interrupting deoxyribonucleic acid (DNA) repair [3-5]. Hyperthermia also helps in reducing resistance to cisplatin by decreasing the mechanisms of cellular resistance [6]. Lastly, it has an immunomodulatory role and improves the immune response against tumor cells by inducing heat shock proteins (HSP), activating antigen-presenting cells, and lymphocyte migration [7].

Although the rising body temperature is associated with significant risks, various methods have been developed for raising the temperature of the intraperitoneal cavity with a minimal increase in the temperature of the rest of the body. During HIPEC, heat is applied loco-regionally and the body’s core temperature is controlled. The anesthesia team aids in core temperature control by applying ice packs in the neck and groin regions. Moreover, automated pumps are available, which are specifically designed for temperature-regulated continuous drug delivery and monitoring of the infusion process.

History

The history of HIPEC is interesting. Egyptian doctors treated tumors with heat around 5000 BC. The Greeks too realized the importance of thermal energy in some form of medical treatment. The most ancient texts of the Law of Moses mention hot springs as having medicinal values. While grazing donkeys, Anah discovered the hot and medicinal springs in the desert, a rare and valuable finding. The techniques in the past have utilized fever-causing microbial extracts or hot water baths for the induction of hyperthermia to treat various ailments [8]. However, both approaches are difficult to monitor. The use of hyperthermia in cancer treatment is first documented by De Kizowitz from France, in 1779. He assessed the effect of fever caused by malaria on malignant tumors. In 1866, Busch in Germany reported the remission of a facial sarcoma in a patient with a high fever due to erysipelas [9]. William Coley, a surgeon from New York Memorial Cancer Hospital, developed the “Coley toxin” in the 1800s, which is a mixture of bacterial culture. It was used to induce hyperthermia in cancer patients. It was the first specialized bacterial antitumor pyrogen [10]. He managed to treat 38 patients with cancers, who had a high-grade fever: 12 patients had complete regression of tumors while 19 patients improved. In 1898, Westermark used total-body hyperthermia with hot baths to treat inoperable cervical cancer and found promising results [11].

In the twentieth century, localized heat was used in the form of galvanocautery to treat cervical and uterine cancers [12]. It was found that cancer cells were more sensitive to heat than normal tissues of the body. These findings were reproduced by others as well. Immersion in heated water is another method evaluated. This was used to achieve localized hyperthermia of the limbs or of the entire body. This was used for disseminated tumors.

Luk et al. (1980) investigated hyperthermia with radiotherapy as a treatment modality in patients with advanced cancers not responding to conventional treatment. It was found that hyperthermia achieved by microwave combined with radiotherapy was found to have a higher tumor regression rate as compared to either modality alone. Luk’s study was a pilot study in which a temperature average of 42.5 degrees was induced by microwave diathermy to treat superficial cancers. The response of recorded tissue temperatures, either alone or in combination with radiation therapy, was evaluated. The treatment was well-tolerated by patients with only a few side effects [13].

HIPEC was first introduced by John Spratt in 1980 [14]. He treated a pseudomyxoma peritonei patient with intraperitoneal thiotepa followed by hyperthermic intraperitoneal methotrexate. Koga et al. confirmed the role of combined hyperthermia and intraperitoneal chemotherapy in the treatment of implanted peritoneal cancer in rats [15]. He showed that optimal control of peritoneal metastasis was achieved not by heat or chemotherapy alone but by a combination of hyperthermia with chemotherapy. Yonemura and Kanazawa established the role of this treatment in the prevention of gastric cancer peritoneal disease and for the treatment of established disease [16]. The role of HIPEC in improving survival outcomes in peritoneal disease is slowly being established in interval debulking surgery, following neoadjuvant chemotherapy, secondary cytoreductive surgery, and, most recently, in the setting of primary cytoreductive surgery.

Candidates for HIPEC

Women with primary and recurrent stage IIIc ovarian cancer are most commonly recruited in various observational and interventional studies evaluating the role of HIPEC. Studies performed in ovarian malignancy stages Ic to IIIc have reported better survival outcomes with the use of HIPEC [17]. Evidence of the use of HIPEC in recurrent ovarian cancer is limited to a single trial and a few retrospective studies [18]. There is no consensus regarding the chemotherapeutic drug to be used, the protocol for agent delivery, and postoperative therapy for HIPEC in recurrent ovarian cancer.

Technique

After resection of all gross disease, the abdominal cavity is initially irrigated with normal saline to remove all particulate matter that may block the outflow circuit and to ensure that there is adequate hemostasis prior to HIPEC delivery. Enteric reconstruction can be carried out prior to or after HIPEC therapy. Hyperthermia can be generated by various perfusion systems that are available. These systems include closed circuit pumps, which deliver heated chemotherapy drugs through inflow catheters with drainage accomplished via outflow catheters. HIPEC can be performed using two techniques, the open or coliseum technique and the closed technique.

Open Technique

In the open technique, a big, nonporous synthetic mesh is sewed to the edges of the skin incision, and the abdomen is tented up using retractors. The surgeon manually stirs the perfusate in the abdomen for even distribution and adequate exposure of organs to heated chemotherapy. Inflow and outflow catheters are located on the lateral abdominal wall. The advantages and disadvantages of the open technique are listed in Table 1.

**Table 1:** Advantages and disadvantages of the open technique [19]

Advantages of the open technique	Disadvantages of the open technique
Even distribution of heated chemotherapy throughout the abdominal cavity	Risk of exposure
	Risk of spillage
	Time-consuming
	Accelerated heat loss

At our center, HIPEC is done as an ‘open coliseum technique.’ A small Bookwalter retractor is used and the skin is sutured to the frame. The frame is fixed about 3 to 4 inches above the abdomen. This ensures that there is no spillage of the drug. Once sutured, the abdomen is closed with a clear X-ray cassette dressing with a small opening made in the dressing so as to allow for mixing of the drug(s). The technician heats the circulating fluid using D5W to 40⁰C to 42⁰C before injecting the chemotherapy drug.

Closed Technique

In the closed technique, after cytoreduction, the inflow and outflow catheters are introduced into the abdominal cavity through the midline incision. The abdominal incision is sutured ensuring a watertight closure. The abdomen is then instilled with the carrier solution following which perfusion begins. Once the goal temperature of 40⁰C-42⁰C is reached, the chemotherapy drug is filled in the reservoir and perfused for 30 to 120 minutes. During the procedure, the abdominal wall may be agitated to facilitate even drug distribution throughout the abdominal cavity. After the perfusion cycle is complete, the chemotherapy drug is drained through the outflow catheter and the abdomen is then irrigated with normal saline. The advantages and disadvantages of the closed technique are mentioned in Table 2.

**Table 2:** Advantages and disadvantages of the closed technique [19]

Advantages of the closed technique	Disadvantages of the closed technique
Minimal heat loss	Unequal intra-abdominal distribution of chemotherapy drugs
Easy achievement of intraperitoneal goal temperature	Higher concentration of drug in the blood, leading to myelosuppression
Reduced chance of spillage and exposure to chemotherapy drugs	
Less time consuming	
The theoretical benefit of improved drug penetration	

Chemotherapy drugs used for HIPEC

An ideal chemotherapy drug to be used for HIPEC should be cell-cycle non-specific, water-soluble, having a synergistic effect with heat, with a high molecular weight and proven cytotoxic effect. There has been no consensus in terms of the choice of chemotherapy drug, its dosage, dwell time, goal temperature, and duration of perfusion. These parameters vary widely across various studies conducted until now as shown in Table 3.

**Table 3:** Studies describing the role of HIPEC in primary ovarian cancer HIPEC: hyperthermic intraperitoneal chemotherapy

Study	Design	Inclusion criteria	Drugs	Results
Lim 2017 [20]	Randomized controlled trial: HIPEC+ cytoreductive surgery (CRS) + systemic chemotherapy (n = 92), CRS + systemic chemotherapy (n = 92)	Primary ovarian cancer stages 3 and 4	Cisplatin 75 mg/m2, 90 min at 41.5⁰C	5-year progression-free survival (p = 0.569): HIPEC: 20.9%, Control: 16%; 5-year overall survival (p = 0.574): HIPEC: 51%, Control: 49.4%
Van Driel 2018 [21]	Multicentric randomized controlled trial: CRS + HIPEC (n = 122), CRS alone (n= 123)	Stage III ovarian cancer patients who have received neoadjuvant chemotherapy with carboplatin and paclitaxel with stable disease after 3 cycles	Cisplatin 100 mg/m2, 90 min at 40⁰C	Progression[Ns1]-free survival: HIPEC: 14.2 months, Control: 10.7 months; Median overall survival: HIPEC: 45.7 months, Control: 33.9 months Adverse events of grade 3 and 4: 25% and 27%
Ansaloni et al. 2012 [22]	Open prospective phase 2 study	Primary or recurrent peritoneal carcinomatosis with ovarian cancer; Primary (n=9), Recurrent (n=30)	Cisplatin 100 mg/m2, Paclitaxel 175 mg/m2, Doxorubicin 35 mg/m2, 90 min at 41.5⁰C, 66% received cisplatin + doxorubicin.	Recurrence rate 59%; Mean recurrence time 14.4 months; Mean hospital stay 23.8 days

In general, cisplatin is the most commonly used chemotherapy drug used for HIPEC in gynecological malignancies and is recommended by the National Comprehensive Cancer Network (NCCN) guidelines. Other commonly used drugs in HIPEC include paclitaxel, liposomal doxorubicin, either alone or in various combinations. In the setting of primary ovarian cancer, Lim et al. and Van Driel described the use of intraperitoneal cisplatin while Ansaloni et al. used intraperitoneal cisplatin, paclitaxel, and doxorubicin in doses described in Table 3 [20-22]. There has been no consensus in terms of the ideal chemotherapy drug regimen in women receiving HIPEC for the treatment of recurrent ovarian cancer. Fagotti described the use of oxaliplatin while Bakrin et al. used cisplatin alone or along with mitomycin C or doxorubicin [17-18]. Spiliotis et al. used cisplatin and paclitaxel in platinum-sensitive women while doxorubicin and paclitaxel or mitomycin C was given to platinum-resistant women [23]. Large randomized controlled trials are needed in the future to address this. Table 4 and Table 5 show various chemotherapeutic agents used in HIPEC and their mean peak peritoneal activity, respectively.

**Table 4:** Drugs used for HIPEC and their dosage HIPEC: hyperthermic intraperitoneal chemotherapy; AUC: area under the curve

Drug	Dosage
Cisplatin	50 -75 mg/m2
Doxorubicin	30- 75 mg/m2
Gemcitabine	1000 mg/m2
Paclitaxel	60 -175 mg/m2
Oxaliplatin	360 – 480 mg/m2
Caelyx	20 – 50 mg/m2
5FU	600 mg/m2
Docetaxel	40 – 150 mg/2
Carboplatin	AUC 5-6

**Table 5:** Mean peak peritoneal cavity: plasma concentration ratio of various chemotherapy agents used in HIPEC HIPEC: hyperthermic intraperitoneal chemotherapy

Carboplatin	18
Cisplatin	20
5FU	298
Paclitaxel	1000
Doxorubicin	494
Mephalan	94
Caelyx	600 – 1000
Gemcitabine	500 – 800

Timing of HIPEC

Intraperitoneal chemotherapy is usually performed immediately after cytoreduction. There is uncertainly on whether the complications are increased if done after any gastrointestinal anastomosis. At our center, HIPEC is preferably performed just prior to closure (after bowel anastomosis, etc). Concerns about the inconveniences of delivery and toxicities associated with postoperative intraperitoneal chemotherapy motivated researchers to work out whether HIPEC could improve safety and quality of life.

Temperature

The synergistic effect of hyperthermia and intraperitoneal chemotherapy is observed from a temperature of 39°C and thereafter increases linearly. However, bowel tissue tolerance to temperature limits the upper limit of heat administered intraperitoneally. A study conducted by Shimizu et al. found that heat administered intra-abdominally in rats was safe at a temperature of 39°C. However, the application of heat at a temperature of 46°C or 45°C leads to a mortality of 100% and 90%, respectively. Also, 100% survival was found at 44°C [24]. Retrospective studies in humans show that an intra-abdominal temperature above 42°C is associated with a higher complication rate [25]. Keeping these data in mind, most surgical oncologists have reached a general consensus of 41°C-43°C as the desired level of intra-abdominal hyperthermia.

Duration

The duration of HIPEC varies from 30 minutes to 120 minutes depending on the institutional protocol and other factors like the pharmacokinetics of chemotherapy drugs, patients' cell count, and renal function.

Role of HIPEC in primary ovarian cancer

The role of HIPEC in the management of primary ovarian carcinoma is still debatable. Most of the studies have not shown any benefit. However, there are a few studies that have shown good results with HIPEC in primary ovarian cancer. Ansaloni et al. (2012) conducted a prospective phase 2 study recruiting 39 patients with primary or recurrent peritoneal carcinomatosis. Seventy-seven percent (77%) of patients had recurrent carcinomatosis and 23% had a primary tumor. Cytoreductive surgery was performed in these patients after which HIPEC was performed. The procedure was performed for 90 minutes, with an intraperitoneal temperature of 41.5°C. Fifty-nine percent (59%) of patients had recurrence with the mean recurrence time being 14.4 months [22].

Lim et al. in 2017 conducted a randomized controlled trial in women with stages 3 and 4 primary ovarian cancer. There was no significant difference found in progression-free survival (PFS) in the HIPEC versus control groups (43.2% vs. 43.5% at 2 years and 20.9% vs. 16.0% at 5 years; P= 0.569). Five-year OS was also found to be similar in both groups [20].

Van Driel et al. conducted a multicentric, prospective randomized controlled trial in women with stage 3 ovarian cancer who had stable disease after receiving three cycles of neoadjuvant chemotherapy. It was a landmark trial in which 245 patients, who underwent interval cytoreductive surgery (CRS) with or without HIPEC, were recruited. A total of 122 patients were randomly assigned to receive intraperitoneal cisplatin (100 mg/m^2^) after an intra-peritoneal temperature of 40°C was achieved with heated saline. One-hundred twenty-three (123) women underwent CRS without HIPEC. Patients received an additional three cycles of adjuvant chemotherapy. The median overall survival rate was 33.9 months and 45.7 months in the surgery and surgery with HIPEC groups, respectively. The median recurrence-free survival rate was 10.7 months in the surgery group and 14.2 months in the surgery with HIPEC group. Grades 3 and 4 toxicities were similar in both groups (25% vs 27% in the surgery and surgery with HIPEC groups, respectively). The drawbacks of this study include the exclusion of stage 4 ovarian cancer [21].

Forty (40) patients with advanced ovarian malignancy, who were planned for upfront cytoreductive surgery along with HIPEC, were enrolled in a phase II, non-randomized, single-arm study. Patients received adjuvant chemotherapy with bevacizumab after surgery. Complete cytoreduction was achieved in all the cases. Early complications were observed in 23 patients, which included hematological toxicity, pleural effusion requiring a drain, relaparotomy for hemorrhage, and bowel anastomosis dehiscence. Eight patients had major complications (pleural effusion with drain and bowel anastomosis dehiscence). Overall, the data suggested that the safety of HIPEC in the upfront treatment of advanced ovarian cancer is reasonable [26]. The major complications reported in this study were pleural effusion requiring a drain in five patients and bowel anastomosis dehiscence in three patients. The reported late complications were mild and related to kidney failure. No postoperative death was reported in the series.

In a recent trial from China, survival outcomes were compared between primary cytoreductive surgery (PCS) with HIPEC versus PCS alone for patients with stage III epithelial ovarian cancer. Four-hundred twenty-five (425; 72.8%) underwent PCS with HIPEC and 159 (27.2%) underwent PCS alone. The median survival time was 49.8 (95%CI, 45.2-60.2) months for patients undergoing PCS with HIPEC and 34.0 (95%CI, 28.9-41.5) months for patients undergoing PCS alone, and the three-year overall survival rate was 60.3% (95%CI, 55.3%-65.0%) for patients undergoing PCS with HIPEC and 49.5% (95%CI, 41.0%-57.4%) for patients undergoing PCS alone. Subgroup analysis was further done in the complete and incomplete surgery subgroups. Patients in the PCS with HIPEC group had significantly better survival than those in the PCS group, except for the three-year overall survival rate in the incomplete subgroup. Among those who underwent complete surgical procedures and comparing those who received PCS with HIPEC vs those who received PCS alone, the median survival time and the three-year overall survival rate were better in the HIPEC group (P = .04). In this study, the PCS with HIPEC treatment approach was associated with better long-term survival [27].

A randomized phase 3 trial showed a significant benefit in recurrence‐free and overall survival when HIPEC was added to interval cytoreductive surgery (CRS) in patients who were not eligible for primary surgery because of the extent of their disease (OVHIPEC trial; NCT00426257). The trial showed no important differences in toxicity or patient‐reported outcomes between the study groups. The extent of surgery and the number of bowel resections were also similar between the two study groups, and the effect of HIPEC was homogeneous across the levels of predefined and post hoc subgroups. Nevertheless, the design and results of the OVHIPEC trial were critically assessed, and this resembles the reluctance to adopt the positive results of earlier intraperitoneal chemotherapy studies [28].

Role of HIPEC in recurrent ovarian cancer

There is no clear consensus as to the usefulness and the protocol most suitable for HIPEC in women with recurrent ovarian cancer. In 2012, in a case-control study conducted by Fagotti et al., performing HIPEC led to a significant reduction in secondary recurrence and mortality of women having recurrent platinum-sensitive ovarian cancer [17].

In a retrospective multicentric study of recurrent or persistent ovarian cancer(n= 246 ), treated by cytoreductive surgery and HIPEC in two French centers, Bakrin et al. (2012) found no significant difference in overall median survival rate in platinum-resistant and platinum-sensitive disease (48 months in platinum-resistant disease and 52 months in platinum-sensitive disease, respectively; p=0.568) [18]. In another retrospective case-control study conducted by Le Brun (2014) recruiting women with first ovarian cancer relapse, significantly improved overall survival was observed in women receiving CRS followed by HIPEC [29].

A case-control study conducted by Safra et al. (2014) in women with recurrent epithelial ovarian cancer also suggested significant improvement in five-year survival and progression-free survival in women undergoing surgery with HIPEC as compared to those receiving systemic chemotherapy alone. Treatment outcome according to the patients' breast cancer gene (BRCA) status was also compared. Fifty-one point two percent (51.2%) women in controls and 51.9% women in the HIPEC group were BRCA gene mutation carriers. BRCA gene mutation carriers were also found to have significantly improved survival on adding HIPEC after CRS (in BRCA gene mutation carriers, progression-free survival was 20.9 months in the HIPEC group and 12.6 months in the chemotherapy alone group, respectively; p=0.048) [30]. An observational study by Classe and Petrillo also showed favorable survival rates in women with recurrent ovarian cancer receiving CRS followed by HIPEC [31-32].

In 2015, Spiliotis et al. conducted the first randomized controlled trial recruiting 120 women with stages IIIc and IV disease who had recurrence after initial treatment with debulking surgery followed by systemic chemotherapy. Group A comprising 60 women was treated with secondary debulking surgery followed by HIPEC and systemic chemotherapy. Thirty-four (34) patients were platinum-sensitive with 26 who were platinum-resistant. Intraperitoneal cisplatin 100 mg/m2 and paclitaxel 175 mg/m2 was administered for 60 minutes at 42.5°C in women with platinum-sensitive disease. Doxorubicin 35 mg/m2 and either paclitaxel 175 mg/m2 or mitomycin 15 mg/m2 was delivered for 60 minutes at 42.5°C. In 40 women, the open technique was used while in 20 women, the closed technique was used. Women in Group B underwent debulking surgery followed by systemic chemotherapy alone. The mean overall survival rate was found to be significantly higher in group A compared to group B (26.7 versus 13.4 months; P <0.006). The three-year survival rate was 75 % in group A compared to 18 % in group B (P <0.01). In platinum-sensitive disease, survival was significantly higher in the HIPEC group compared to the non-HIPEC group (26.8 versus 16.2 months; P=0.035). In platinum-resistant disease, no significant difference was observed in survival rate in the HIPEC group and the non-HIPEC group (26.6 months versus 10.2 months). Survival was 30.9 months in women who received HIPEC with CC-0. Survival at CC-1 and CC-2 in the HIPEC group was 23.9 months and 12.1 months, respectively. These were statistically different when compared to the non-HIPEC group, in which survival was 16.1 months with CC-0, 11 months with CC-1, and 6.7 months with CC-2(P=0.02) [22].

However, Baiocchi et al. in a retrospective observational study found no significant improvement in survival rate with the addition of HIPEC to secondary cytoreductive surgery. HIPEC was associated with a significantly higher grade III and IV morbidity [33].

Gynecologic Oncology Group-0213 (GOG-0213) is an international, open-label, randomized phase 3 trial in women with platinum-sensitive ovarian cancer in which they showed that secondary cytoreduction did not improve OS. However, this trial has some pitfalls like the compromise of the prespecified stratification variable [34]. In this trial, there were no defined patient eligibility criteria for surgery, and eligibility was based on the surgeon's preference. There was a lack of uniformity in selection criteria or in the method-defined surgical technique across the various participating centers. The only requirements for GOG-0213 enrollment were platinum-sensitive recurrent ovarian cancer with the possibility of achieving a complete gross resection (CGR) and good medical condition, with acceptable kidney, liver, and bone marrow function, as well as a GOG performance status score of 0-2. The decision to perform surgery was at the surgeon's discretion. Their data also lacked information on the extent of residual disease after primary debulking surgery and the site of recurrence in some patients. Ascites was mentioned as an exclusion criterion, but it was not further specified if any ascites or a threshold of a certain amount of ascites was exclusive and how much ascites a patient had at diagnosis. To overcome these pitfalls, the DESKTOP (Descriptive Evaluation of preoperative Selection KriTeria for Operability) I trial was planned.

The DESKTOP I was a trial that evaluated a score for the prediction of complete cytoreduction in recurrent ovarian cancer. Resectability at recurrent cancer was improved if three factors, i.e. complete resection at first surgery, good performance status, and absence of ascites, were present [35]. The DESKTOP II trial verified this hypothesis prospectively in a multicenter setting [36]. The DESKTOP III trial investigated the role of surgery in recurrent platinum-sensitive ovarian cancer. A total of 407 patients with recurrent ovarian cancer and a first relapse after a six-month or longer platinum-free interval were randomized to the surgery with adjuvant chemotherapy (n = 206) or only chemotherapy without surgery (n = 201) groups. This was a superiority trial, with overall survival (OS) as the primary endpoint. Median OS in the intent-to-treat population was 53.7 months with and 46.0 months without surgery (hazard ratio 0.75, 95% confidence interval 0.58-0.96; P = .02). Median PFS was 18.4 months with surgery and 14.0 months without (hazard ratio 0.66, 95% confidence interval 0.54-0.82; P < .001). DESKTOP III is the first prospectively randomized trial showing an OS benefit of debulking surgery in recurrent ovarian cancer. SOC-3 (Surgery and Niraparib in Secondary Recurrent Ovarian Cancer) is an ongoing multicenter, randomized controlled, phase II trial of secondary cytoreduction followed by chemotherapy and niraparib (PARP-inhibitor) maintenance versus chemotherapy and niraparib maintenance in patients with platinum-sensitive, second relapsed ovarian malignancy. With the above-mentioned completed trials, the role of secondary cytoreduction is well-proven. The addition of HIPEC with secondary cytoreduction needs further validation [37].

Various trials are ongoing in this field including the HORSE (Chemotherapy (HIPEC) in Ovarian Cancer Recurrence) and CHIPOR (Hyperthermic Intra-Peritoneal Chemotherapy (HIPEC) in Relapse Ovarian Cancer Treatment) trials [38-39]. The HORSE trial was a multicentric randomized controlled trial in which progression-free survival will be compared between women having first ovarian cancer recurrence, receiving surgery plus HIPEC versus surgery alone [38]. Similarly, in the CHIPOR trial, 444 patients with ovarian cancer recurrence were recruited and randomized to either undergo surgery with HIPEC or surgery alone [39]. Table 6 describes the role of HIPEC in recurrent ovarian cancer.

**Table 6:** Studies describing the role of HIPEC in recurrent ovarian cancer HIPEC: hyperthermic intraperitoneal chemotherapy

Study	Design	Inclusion criteria	Drugs	Results
Fagotti 2012 [17]	Case-control study: 30 cases CRS + HIPEC, 37 controls CRS alone	Platinum-sensitive recurrent ovarian cancer	Oxaliplatin 460 mg/m2, 41.5⁰C for 30 min, closed technique	Secondary recurrence 66.6% in cases, 100% in controls (P=0.001)
Bakrin 2012 [18]	Retrospective multicentric study (n=246)	Recurrent and persistant ovarian cancer treated with optimal CRS + HIPEC; Platinum-resistant persistant (n=62); Platinum-sensitive recurrent (n=184)	95.5% received cisplatin alone or in combination with mitomycin C or doxorubicin, 90 minutes at 44⁰C-46⁰C, open and closed technique	Overall median survival: 48 months in platinum-resistant and 52 months in platinum-sensitive (P=0.568)
Spiliotis 2015 [23]	Randomized controlled trial: Secondary CRS + HIPEC + systemic chemotherapy (n= 60), Secondary CRS + systemic chemotherapy (n=60)	Stage IIIc & IV ovarian cancer with recurrence after debulking surgery followed by systemic chemotherapy	Platinum-sensitive: cisplatin 100 mg/m2 + paclitaxel 175 mg/m2; Platinum-resistant: doxorubicin 35 mg/m2 + paclitaxel 175 mg/m2 or mitomycin 15 mg/m2 60 min at 42.5⁰C; Open technique (n=40), Closed technique (n=20)	Mean overall survival: HIPEC: 26.7 months, No HIPEC: 13.4 months (P<0.006)
Le Brun 2014 [29]	Retrospective case-control study: CRS + HIPEC (n = 23), CRS alone (n =19)	Women with first ovarian cancer relapse receiving second-line chemotherapy followed by CRS	Cisplatin (n= 16): 16 mg/m2, eloxatin (n=6): 6 mg/m2, mitomycin C (n=1): 1 mg/m2, 42⁰C for 1 hour for cisplatin and 30 min for eloxatin and mitomycin C.	4-year overall survival: CRS + HIPEC: 75.6%, CRS alone: 19.4 % (P=0.013)
Safra 2014 [30]	Case-control study: CRS+HIPEC (n=27), systemic chemotherapy alone (n= 84 )	Recurrent epithelial ovarian cancer who underwent BRCA gene mutation testing	Cisplatin 50 mg/m2 and doxorubicin 15 mg/m2, Paclitaxel 60 mg/m2 and carboplatin, Cisplatin 25 mg/l/m2 and mitomycin-C 3.3 mg/l/m2 120 min at 42.5 ⁰C	Median progression-free survival rate: HIPEC: 15 months systemic chemotherapy: 6 months (P=0.001). 5-year survival rate 79% in CRS + HIPEC, 45% in systemic chemotherapy (P=0.016)
Baiocchi 2015 [33]	Retrospective observational study: Secondary cytoreduction alone (n=50), Secondary cytoreduction + HIPEC (n=29)	Platinum-sensitive recurrent ovarian cancer undergoing secondary cytoreduction	Mitomycin C (10 mg/m2) and cisplatin (50 mg/m2), Cisplatin (50 mg/m2) and doxorubicin (n=8), Cisplatin alone (50 mg/m2) (n=3), Closed technique 41⁰C-42⁰C for 90 min	Median overall survival: no HIPEC: 59.3 months, secondary cytoreduction with HIPEC: 58.3 months (P=0.95); Median disease-free survival: no HIPEC: 18.6 months, HIPEC + surgery: 15.8 months (P=0.82)

Consensus guidelines

Due to these contradictory results of HIPEC, most of the guidelines do not recommend HIPEC as first-line therapy. As per ESMO guidelines, HIPEC is not considered a standard of care as first-line treatment and its use should be limited to well-designed RCTs [40]. Vergote et al. also concluded that HIPEC should not be considered as the standard of care or first-line treatment in the management of ovarian cancer [41]. Because of the positive results by Van Driel [20] and other prospective studies, the NCCN recommends HIPEC with cisplatin (100 mg/m2) to be considered as an option following interval debulking surgery (IDS) in women with stage 3 ovarian cancer who responded or had stable disease after three cycles of neoadjuvant chemotherapy (NACT) [42].

Safety of HIPEC in ovarian cancer

The morbidity of HIPEC, when combined with cytoreduction, is mainly due to surgical complications such as anastomotic leaks, intra-abdominal hemorrhage, and sepsis. Toxicities specific to HIPEC are mainly hematological and renal. Transient bone marrow suppression, anemia, leukopenia, and thrombocytopenia are the frequently reported hematological complications. Acute kidney injury is the most common toxicity in patients undergoing HIPEC with cisplatin. Cisplatin-associated nephrotoxicity can be prevented by using nephroprotectants such as sodium thiosulphate although larger studies are needed to determine the choice of and the optimal dose of nephroprotectants. Other adverse effects are specific to the chemotherapy drugs used. Oxaliplatin is associated with bleeding complications. HIPEC with cytoreductive surgery is associated with higher chances of toxicity as compared to cytoreduction alone. However, recent studies have shown that the toxicity rate of HIPEC alone and HIPEC combined with cytoreduction are similar. In the multicentric RCT conducted by Van Driel, the grade 3-4 toxicity rate was similar in both the groups (25% versus 27% in cytoreduction alone and cytoreduction with HIPEC, respectively) [20]. In the retrospective study conducted by Bakrin, overall morbidity was 31% and mortality was 0.5%. Leukopenia, intra-abdominal hemorrhage, and an anastomotic leak occurred in 11.6%, 3%, and 2.4% respectively [18].

ERAS in HIPEC

Enhanced Recovery After Surgery (ERAS) Society recommendations, 2020, have provided guidelines for ERAS in cytoreductive surgery with and without HIPEC (CRS±HIPEC) [43]. It is recommended that preoperative counseling should be indicated routinely to improve quality of life, somatic symptoms, and psychological outcomes. Preoperative nutritional screening by the use of a validated tool and by measuring serum albumin is also recommended. In patients with malnutrition or at risk for malnutrition, nutritional and protein (>1.2 g/kg/day) supplementation (oral>enteral>parenteral) for at least five days and up to 14 days in cases of severe malnutrition is recommended routinely. In these surgical candidates, a combination of at least two antiemetic drugs (ondansetron, dexamethasone, droperidol) should be indicated routinely to prevent postoperative nausea and vomiting. Prophylactic antibiotics within one hour before incision for CRS±HIPEC without the need for routine repeated administration should be indicated routinely to prevent surgical site infection. Total intravenous anesthesia as an alternative to inhalation anesthesia could be indicated to prevent postoperative nausea and vomiting. Preoperative mechanical bowel preparation alone for patients undergoing CRS±HIPEC including probable colectomy should not be indicated to reduce the incidence of surgical site infection and an anastomotic leak. Preoperative mechanical bowel preparation is indicated with probable rectal resection. Epidural analgesia (T5-T11, low dose of local anesthetic and opioids) for 72 h after CRS/HIPEC should be indicated routinely to obtain pain relief, spare opioids, and hasten the resumption of bowel function. Limiting postoperative fluid-related weight gain (target: < 3.5 kg on postoperative day 3) is advised.

Future perspectives

Most ongoing trials are being conducted on women having primary ovarian cancer. Cisplatin is the most commonly used HIPEC drug in these trials followed by paclitaxel. In the HIPECOV trial, women with both primary and recurrent ovarian cancer are being recruited and the efficacy of HIPEC with lobaplatin following CRS will be evaluated. HORSE and CHIPOR are two large trials that are being conducted, recruiting women with recurrent ovarian cancer. In these two trials, women would undergo platinum-based NACT followed by interval debulking surgery followed by HIPEC in the treatment arm [37-38]. Until now there is no standard guidelines with respect to the exact patient population and the histology of ovarian tumor in which HIPEC would prove to be most beneficial. There is, at present, no standardized chemotherapy drug regimen or guidelines on treatment duration and temperature decided for performing HIPEC. These ongoing trials could probably provide answers to these questions, and their results are eagerly awaited.

Other controversial issues are whether HIPEC is effective as an addition to upfront surgery and after surgery for recurrent disease, whether it has better efficacy as compared to adjuvant intraperitoneal chemotherapy, what are the side/adverse effects of hyperthermia on the body, what is the optimal health care setting required to perform HIPEC, and the use of biomarkers for selecting a suitable subset of patients likely to benefit from HIPEC.

Pressurized intraperitoneal aerosol chemotherapy (PIPAC) is a novel technique in which the chemotherapy drug is delivered in an aerosol form inside the abdominal cavity and maintained at high pressure inside the abdominal cavity. The aerosol form leads to even distribution of the drug on the peritoneal surface while the pressure ensures deeper penetration of the drug. During laparoscopy, the chemotherapy drug is delivered from a high-pressure injector (1500 kPa) through a micropump into the abdominal cavity, and after 30 minutes, the drug-aerosol is suctioned out through a suction system [44]. PIPAC and its role is being explored in women with recurrent ovarian cancer with peritoneal metastasis. Women with peritoneal metastasis are candidates for systemic palliative chemotherapy. However, systemic chemotherapy is less effective in peritoneal metastasis due to poor tumor absorption. In such women, intraperitoneal chemotherapy with PIPAC without cytoreductive surgery has been found to be safe and well-tolerated [45]. It allows for improved tumor penetration and intra-abdominal dissemination. In a systematic review conducted by Tempfer et al., the objective tumor response rate was 69%, and the mean overall survival duration was 13.7 months. It was concluded that PIPAC maintained the quality of life and was found feasible safe and effective in women with ovarian cancer and peritoneal carcinomatosis [46]. In a single-arm, phase 1, nonrandomized study, PIPAC with dose-escalating cisplatin and doxorubicin could safely be applied in women with recurrent ovarian cancer [47]. All studies conducted are single-armed studies and large randomized control trials are needed to provide further evidence in favor of this novel technique.

Table 7 summarizes all ongoing trials studying the role of HIPEC in ovarian cancers.

**Table 7:** Ongoing trials on HIPEC [38-39], [48-50] HIPEC: hyperthermic intraperitoneal chemotherapy; CRS: cytoreductive surgery; PFI: progression-free interval; OS: overall survival; NACT: neoadjuvant chemotherapy; DFS: disease-free survival; IDS: interval debulking surgery; EOC: epithelial ovarian cancer

NCT number	Trial acronym	Trial title	n	Indication	Treatment arm	Control arm	HIPEC drug	Duration of trial	Country	Outcome
NCT01539785	HORSE	Surgery plus hyperthermic intra-peritoneal chemotherapy (HIPEC) versus surgery alone in patients with platinum-sensitive first recurrence of ovarian cancer: a prospective randomized multicenter trial	158	Recurrent ovarian cancer	CRS with HIPEC	CRS without HIPEC	Cisplatin at 75 mg/m²	September 2012	Rome, Italy	PFI
NCT01376752	CHIPOR	Hyperthermic intraperitoneal chemotherapy (HIPEC) in relapsed ovarian cancer treatment	444	Recurrent platinum-sensitive ovarian cancer	Platinum-based NACT × 6 cycles, followed by CRS with HIPEC	Platinum-based NACT × 6 cycles, followed by CRS without HIPEC	Cisplatin at 75 mg/m²	4/2011– 4/2025	Belgium/France	OS
NCT03842982	CHIPPI	Hyperthermic intraperitoneal chemotherapy (HIPEC) in ovarian cancer	432	Primary EOC	CRS or IDS with HIPEC	CRS or IDS without HIPEC	Cisplatin 100 mg/m2 × 90 min	4/2019– 6/2024	France	DFS
NCT02681432	HIPEC-OVA	Hyperthermic intraperitoneal chemotherapy with paclitaxel in advanced ovarian cancer	60	Primary EOC	CRS with HIPEC	CRS without HIPEC	Paclitaxel 175 mg/m2 × 60 min	1/2012– 12/2019	Spain	OS
NCT03371693	HIPECOV	Cytoreductive surgery (CRS) plus hyperthermic intraperitoneal chemotherapy (HIPEC) with lobaplatin in advanced and recurrent epithelial ovarian cancer	112	Primary and recurrent EOC	CRS with HIPEC	CRS without HIPEC	Lobaplatin 30 mg/m2 at 60’C	9/2017– 12/2020	China	OS

## Conclusions

HIPEC is an alternative to cytoreductive surgery alone and palliative chemotherapy in women with advanced ovarian cancer with extensive peritoneal metastasis. There exists controversy around HIPEC for both primary and recurrent ovarian cancer, and there is a strong need for more randomized controlled trials on this issue to reach some necessary conclusions. There are difficulties in including HIPEC as a standard of care due to the variation and non-standardization of different studies, in design and protocol. Larger, well-designed trials are, therefore, needed to provide further answers in terms of exact protocol and drug regimen that would be suitable to perform HIPEC. The suitability of candidates for HIPEC is still an enigma, and the results of ongoing trials would hopefully provide further knowledge in this direction. The five-year survival of advanced ovarian cancer is around 50% and HIPEC could be a game-changer in the optimal management of women with ovarian cancer and peritoneal metastasis, provided further, well-designed studies prove so.
